# Synthesis of dihydrophenanthridines by a sequence of Ugi-4CR and palladium-catalyzed intramolecular C-H functionalization

**DOI:** 10.3762/bjoc.4.10

**Published:** 2008-04-08

**Authors:** Florence Bonnaterre, Michèle Bois-Choussy, Jieping Zhu

**Affiliations:** 1 Institut de Chimie des Substances Naturelles, CNRS, 91198 Gif-sur-Yvette Cedex, France

## Abstract

**Background:**

Small polyfunctionalized heterocyclic compounds play important roles in the drug discovery process and in the isolation and structural identification of biological macromolecules. It is expected that ready access to diverse sets of heterocycles can not only help improving the known biological and pharmacokinetic properties of drugs, but also assist the discovery of molecules that exhibit biological effects beyond those associated with previously known macromolecules. By virtue of their inherent convergence, high productivity, their exploratory and complexity-generating power, multicomponent reactions (MCRs) are undoubtedly well suited for creating molecular diversity. The combination of MCRs with an efficient post-functionalization reaction has proven to be an efficient strategy to increase the skeleton diversity.

**Results:**

The Ugi reaction of an *o*-iodobenzaldehyde (**2**), an aniline (**3**), an isocyanide (**4**), and a carboxylic acid (**5**) afforded α-acetamido-α-phenylacetamide (**6**) in good to excellent yields. The palladium-catalyzed intramolecular C-H functionalization of these adducts under ligandless conditions provided the functionalized dihydrophenanthridines (**1**).

**Conclusion:**

Highly functionalized dihydrophenanthridines are synthesized in only two steps from readily accessible starting materials in good to excellent overall yields.

## Introduction

**Scheme 1:**
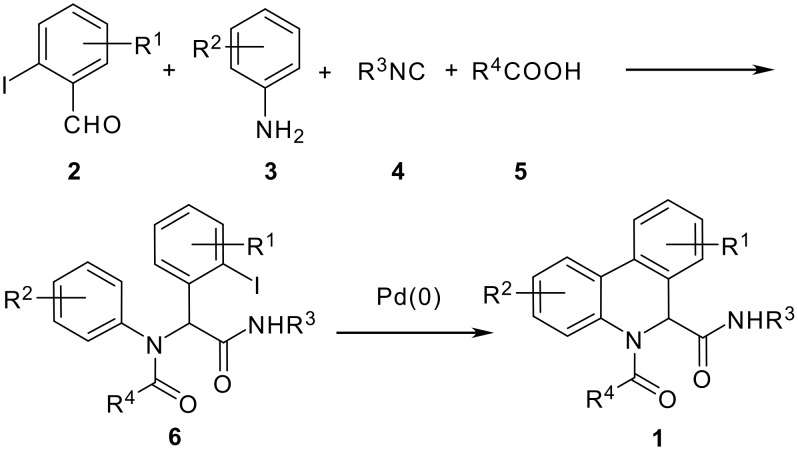
Two-step synthesis of dihydrophenanthridines **1.**

Multicomponent reactions (MCRs) offer a unique way to generate efficiently libraries of complex molecules with high degree of diversity [1–2]. Among them, the Ugi four component reaction (Ugi-4CR) is without doubt one of the most powerful transformations that has been extensively investigated for the past twenty years [3]. In order to further increase its versatility and the complexity-generating power, a variety of reaction types have been associated with Ugi-4CRs for the synthesis of medicinally relevant heterocycles [4–6]. Using this MCR/post functionalization strategy, we have developed two-step syntheses of a number of macrocycles including cyclophanes and cyclodepsipeptides [7–10]. We have also reported the elaboration of Ugi-adduct containing two arylhalide functions for the synthesis of 1,4-benzodiazepine-2,5-diones [11] and their tetracyclic derivatives [12] featuring a key intramolecular C-H functionalization reaction [13–14]. As a continuation of this research program, we were interested in the synthesis of dihydrophenanthridines **1** by combined use of Ugi-4CR and a palladium-catalyzed direct CH-arylation process. The synthetic sequence we envisioned is shown in Scheme 1. The Ugi four-component reaction between an *o*-iodobenzaldehyde **2**, an aniline **3**, an isocyanide **4** and a carboxylic acid **5** should afford an α-acetamido-α-phenylacetamide **6**, which upon palladium-catalyzed C-H activation process should provide dihydrophenanthridine **1** [15–21]. Parallel to our work, Chen, Yang and co-workers have independently developed a similar strategy for the synthesis of this type of heterocycle [22]. In their work, Chen and Yang demonstrated the importance of ligand structure on the outcome of the cyclization [optimized conditions: Pd(OAc)_2_, PCy_3_, K_2_CO_3_, Bu_4_NBr, DMF, 100 °C]; we found that the transformation of **6** to **1** can be realized under ligandless condition using palladium chloride as a palladium source [PdCl_2_, KOAc, DMF, 110 °C] to afford the title compound in good to excellent yield. Furthermore, the presence of activating groups in arenes is not needed for the success of the cyclization. We report herein our results on the two-step synthesis of dihydrophenanthridines **1** [23].

## Results and Discussion

Reaction of *o*-iodobenzaldehyde (**2a**, R^1^ = H), aniline (**3a**, R^2^ = H), *tert*-butyl isocyanide (**4a**) and butyric acid (**5a**) in trifluoroethanol (TFE) at room temperature afforded the four-component adduct **6a** (R^1^ = R^2^ = H, R^3^ = *tert*-butyl, R^4^ = *n*-propyl) in 84% yield. Cyclization of **6a** was examined under a variety of conditions and the results are summarized in Table 1. Treatment of **6a** under conditions we developed previously for the synthesis of tetracyclic compounds that involved a CH-arylation step [DMSO, Pd(OAc)_2_, KOAc, 110 °C] afforded compound **1a** in 58% yield (entry 1) [11–12]. Using other bases such as Ag_2_CO_3_, K_3_PO_4_ and Cs_2_CO_3_ under otherwise identical conditions gave inferior results (entries 2–4). Solvent played also an important role and among those screened (entries 5–9), DMF stood out to afford **1a** in 78% yield (entry 7). We next changed the palladium source and found that PdCl_2_ gave superior results (entry 11). Addition of triphenylphosphine turned out to be beneficial, increasing slightly the yield of **1a** (entries 10, 14), whereas the presence of dppf and P(*o*-tol)_3_ gave reduced yields of the desired compound [24]. Overall, following conditions: PdCl_2_, DMSO, KOAc, 110 °C turned out to be optimal for the present CH arylation process in terms of the yield and simplicity of the manipulation (entry 11). It is interesting to note that the alternative reaction pathway leading to the formation of oxindole via an intramolecular N-arylation process was not observed under these conditions [25–27]. The preferential formation of compound **1a** indicated that cyclization via palladium-catalyzed CH functionalization could be, under appropriate conditions, a kinetically fast process related to other elementary reactions [28–34]. These results indicated that by careful choice of reaction conditions, one can easily reach two completely different scaffolds from the same starting material. Such reagent-dependant divergent synthesis of heterocycles is evidently valuable in diversity oriented synthesis.

**Table 1:** Optimization of the direct CH arylation process.

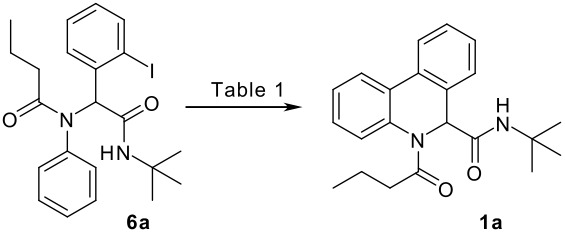						
**Entry**	**Solvent**^a^	**Catalyst**	**Base**	**T °C**	**Time**	**Yield %**

1	DMSO	Pd(OAc)_2_	KOAc	110	23 h	58
2	DMSO	Pd(OAc)_2_	Ag_2_CO_3_	110	6 d	50
3	DMSO	Pd(OAc)_2_	K_3_PO_4_	110	27 h	30
4	DMSO	Pd(OAc)_2_	Cs_2_CO_3_	110	>7 d	nd
5	toluene	Pd(OAc)_2_	KOAc	110	74 h	40
6	dioxane	Pd(OAc)_2_	KOAc	110	74 h	28
7	DMF	Pd(OAc)_2_	KOAc	110	7 h	78
8	DMA	Pd(OAc)_2_	KOAc	110	4 h	56
9	DMA	Pd(OAc)_2_	KOAc	140	3 h	30
10	DMF	Pd(OAc)_2_/PPh_3_	KOAc	110	22 h	82
11	DMF	PdCl_2_	KOAc	110	3 h	83
12	DMF	PdCl_2_(dppf)	KOAc	110	4 h	71
13	DMF	PdCl_2_{P(*o*-tol)_3_}_2_	KOAc	110	>1 d	nd
14	DMF	PdCl_2_/PPh_3_	KOAc	110	3 h	86

^a^ General conditions: concentration of **6a**: 0.01M; 0.5 equiv of catalyst, 2 equiv of base.

The scope of this cyclization was next examined. From three aldehydes, eight amines, five carboxylic acids, and three isocyanides, Ugi-adducts were prepared and their subsequent palladium-catalyzed cyclization was investigated. The results are summarized in Table 2. 4-Nitroaniline (**3c**) is known to be inactive in Ugi reaction due to the reduced nucleophilicity of the nitrogen and the low basicity of the resulting imine leading consequently to a low concentration of the iminium ion. However, by performing the reaction in trifluoroethanol (TFE) [35], the reaction of **3c** with *o*-iodobenzaldehyde (**2a**), *tert*-butyl isocyanide (**4a**) and butyric acid (**5a**) or *N*-(*tert*-butoxycarbonyl)glycine (**5c**) afforded the corresponding Ugi adducts **6c** and **6f** in yields of 31% and 51%, respectively (entries 3, 6). The intramolecular C-H arylation process was found to be insensitive to electronic properties of the aromatic ring. Thus, palladium-catalyzed cyclization of **6b**, **6c**, **6g**, **6h**, bearing electron-donating or electron-withdrawing groups, afforded the corresponding cyclized products in comparable yields (entries 2, 3, 7, 8). Functional groups such as ester, carbamate, ether, heterocyclic nuclei such as pyridine **1i** are tolerated. The aryl chloride function survived under the present conditions leading to dihydrophenanthridine **1g** and **1k** in yields of 74% and 88% (two regioisomers), respectively. The presence of a chlorine atom in **1g** and **1k** provided a handle for further functionalization taking advantage of transition metal catalyzed transformation of aryl chlorides [36]. When Ugi adducts **6j** and **6k** (entries 10, 11) were subjected to the CH-arylation procedure, two regioisomers were produced. Interestingly, in the case of **6k**, a sterically more hindered isomer (**1k-b**) was produced preferentially (entry 11). The cyclization of **6l** could in principle provide a 6-membered as well as a 7-membered ring [37], however, only the cyclization leading to the 6-membered ring occurred to provide **1l** in 80% yield. Finally, cyclization of **6i** gave only one regioisomer **1i** resulting from the activation of C_4_-H, rather than the C_2_-H, of the pyridine. The more pronounced acidity of the C_4_-H could account for the observed regioselectivity [38].

**Table 2:** Two step synthesis of dihydrophenanthridines **1**.

**Entry**	**Aldehyde 2**	**Aniline 3**	**Isocyanide 4**	**Acid 5**	**6 (%)**	**1 (%)**

1	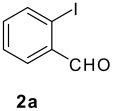	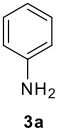	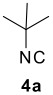	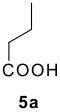	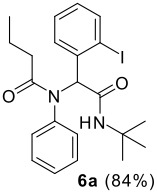	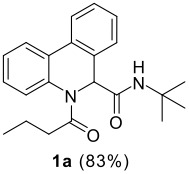
2	**2a**	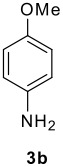	**4a**	**5a**	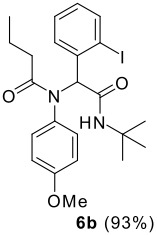	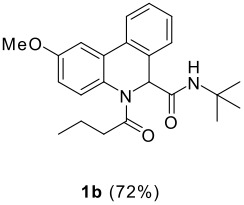
3	**2a**	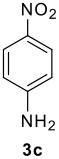	**4a**	**5a**	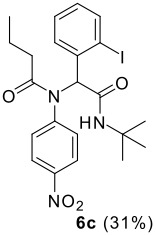	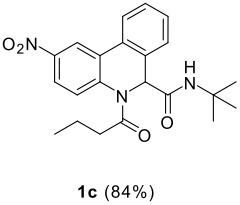
4	**2a**	**3b**	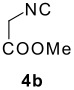	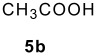	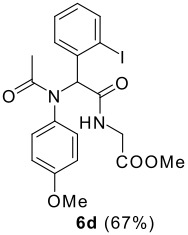	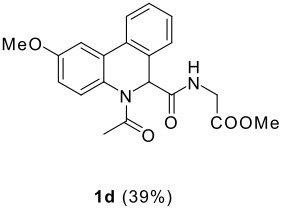
5	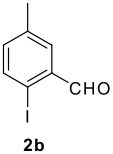	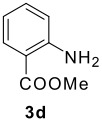	**4a**	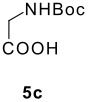	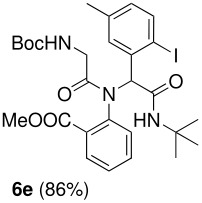	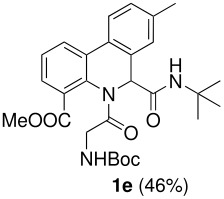
6	**2a**	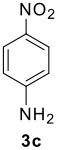	**4a**	**5c**	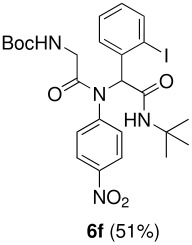	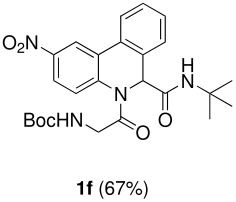
7	**2a**	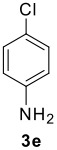	**4a**	**5a**	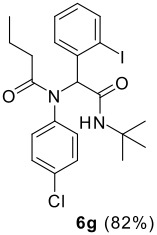	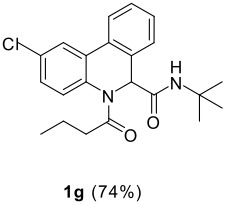
8	**2a**	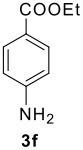	**4a**	**5b**	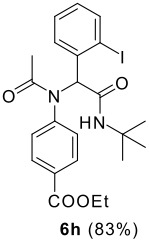	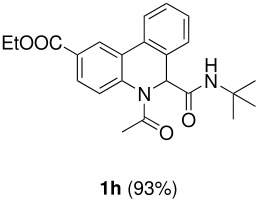
9	**2a**	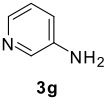	**4a**	**5a**	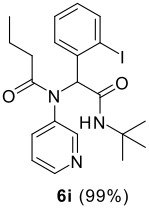	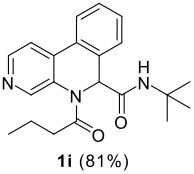
10	**2a**	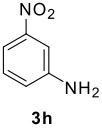	**4a**	**5a**	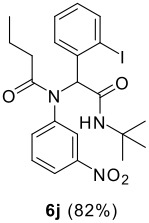	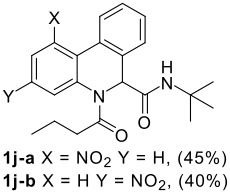
11	**2b**	**3h**	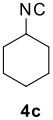	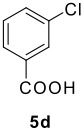	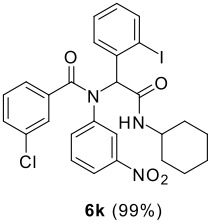	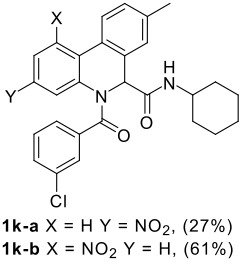
12	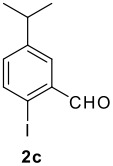	**3a**	**4c**	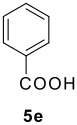	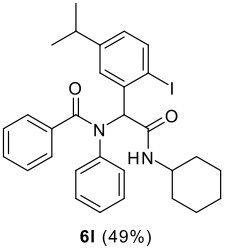	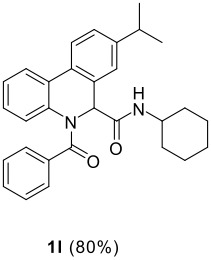

The σ-H bond metathesis has been proposed as one of the major pathways to explain the palladium-catalyzed CH activation process. According to this mechanism, the CH-activation process would depend on the acidity of the CH to be activated rather than the electron density of the aromatic ring and those protons with higher acidity should be energetically more prone to be functionalized. This trend is indeed observed in the present study. The presence of a nitro group in the Ugi adduct did not hamper the reaction, it actually accelerated the CH functionalization reaction. Furthermore, when two protons with similar steric environment were present, the more acidic one is activated selectively as it is observed in the cyclization of **6i** (entry 9).

In conclusion, we have developed an efficient palladium-catalyzed intramolecular CH-arylation reaction leading, under ligandless conditions, to dihydrophenanthridines in good to excellent yields. In combination with the Ugi four-component reaction, this medicinally important heterocycle can be easily prepared in two steps from readily accessible starting materials.

## Experimental

See Supporting Information File 1 for full experimental data.

## Supporting Information

File 1 General information, typical procedure and spectroscopic data of **1a**–**1l.**
