# Supplementary material for: Synthesis of dihydrophenanthridines by a sequence of Ugi-4CR and palladium-catalyzed intramolecular C-H functionalization
Source: Beilstein J Org Chem. 2008 Apr 8;4:10. doi: 10.3762/bjoc.4.10 (PMC2395297; doi:10.3762/bjoc.4.10)
Supplement: File 1 — General information, typical procedure and spectroscopic data of 1a–1l. [file Beilstein_J_Org_Chem-04-10-s001.pdf]

Supporting Information

Synthesis of dihydrophenanthridines by a sequence of Ugi-4CR and palladium-catalyzed intramolecular C-H functionalization

Florence Bonnaterre, Michèle Bois-Choussy, Jieping Zhu*

Institut de Chimie des Substances Naturelles, CNRS, 91198 Gif-sur-Yvette Cedex, France

e-mail: florence.bonnaterre@syngenta.com; Michele.Choussy@icsn.cnrs-gif.fr; zhu@icsn.cnrs-gif.fr

S2: General information

S3: Typical procedure

S3-S10: Spectroscopic data of **1a-1l**

General information

Melting points were recorded using Reichert melting point apparatus.

Mass spectra were obtained either from an AEI MS-9 using electron spray (ES), or from a MALDI-TOF type of instrument for the high resolution mass spectra (HRMS).

Proton NMR (^{1}H) spectra were recorded at 500 MHz or 300 MHz. Carbon NMR (^{13}C) spectra were similarly recorded at 125 or 75 MHz, using a broadband decoupled mode with the multiplicities obtained using a JMOD or DEPT sequence.

Chemical shifts (δ) are reported in parts per million (ppm) from tetramethylsilane. NMR experiments were carried out in $CDCl_3$, CD_3OD or $(CD_3)_2SO$. The following abbreviations are used for the multiplicities: s: singlet, d: doublet, t: triplet, q: quartet, m: multiplet, bs: broad singlet for proton spectra. Coupling constants (J) are reported in Hertz (Hz).

Infrared spectra were recorded on a Perkin Elmer Spectrum BX FT-IR spectrometer.

Flash chromatography was performed using Kieselgel Si 60, 40-63 μm particle sized silica gel (200-400 mesh). Visualization was achieved under a UVP mineralight UVGL-58 lamp, and by developing plates with ninhydrin in butanol/sulfuric acid solution.

Organic solvents were routinely dried and/or distilled prior to use and stored over molecular sieves under argon. All reactions requiring anhydrous conditions were performed in flame-dried apparatus under an argon atmosphere.

All reagents are obtained from commercial suppliers unless otherwise stated.

Experimental section:

Typical procedure for C-H activation of iodoaryl substrates: To a solution of **6a** (0.1 mmol) in DMF (*C* 0.015M) was added KOAc (0.20 mmol), $PdCl_2$ (5 mmol%). The reaction mixture is heated at 110°C under Argon for 3 hours. After cooling the reaction mixture to room temperature, the catalyst and salt were removed by filtration through a short pad of celite. The filtrate was concentrated to dryness and purified through preparative TLC (eluant: Heptane/EtOAc 55:45) to afford **1a** (83%).

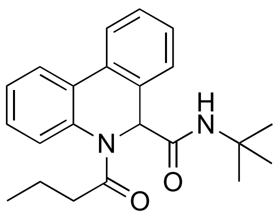

***N-tert*-butyl-5-butyryl-5,6-dihydrophenanthridine-6-carboxamide (1a):** beige solid; mp: 128-130°C; ^{1}H NMR (300 MHz, $CDCl_3$, 298K) (rotamers) δ 0.86 (t, 3H, *J*= 7.0 Hz), 1.13 (s, 9H), 1.55-1.80 (m, 2H), 2.40-2.50 (m, 1H), 2.60-2.70 (m, 1H), 5.76, 6.30 (2 brs, 1H), 7.25-7.50 (m, 7H), 7.80-7.83 (m, 2H); ^{13}C NMR (75 MHz, $CDCl_3$, 298K) δ 13.7, 19.2, 28.6 (3C), 35.9, 51.3, 123.4, 124.9, 125.2, 126.6, 127.9, 128.2, 128.6, 128.8, 130.3, 130.5, 130.7, 135.2, 167.7, 173.5; IR (CH_2Cl_2) ν 3346, 2962, 1669, 1515, 1439, 1366, 1217 cm^{-1}. HRMS *m/z* calcd for $C_{22}H_{26}N_2O_2$ 373.1892 $(M+Na)^+$, found 373.1898.

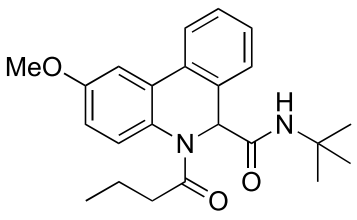

***N-tert*-butyl-5-butyryl-2-methoxy-5,6-dihydrophenanthridine-6-carboxamide (1b):**

72%. Orange oil; ^{1}H NMR (300 MHz, CD_3OD, 298K) (rotamers) δ 0.72-0.78 (m, 3H), 1.14 (s, 9H), 1.50-1.58 (m, 2H), 2.47-2.64 (m, 2H), 3.91 (s, 3H), 6.35 & 6.61 (2brs, 1H), 6.88 & 6.99 (2dd, 1H, *J*= 8.0 Hz, *J*= 2.5 Hz), 7.38-7.50 (m, 5H), 7.90 (d, 1H, *J*= 8.0 Hz); ^{13}C NMR (75 MHz, CD_3OD, 298K) δ 13.9, 19.9, 28.7 (3C), 36.7, 52.6, 56.1, 59.2, 110.6, 114.9, 123.1, 124.9, 128.4, 129.3, 129.5, 129.7, 130.1, 131.7, 132.4, 160.0, 170.3, 175.7; IR (CH_2Cl_2) ν 3326, 2960, 1661, 1613, 1500, 1440, 1390, 1220, 1038 cm^{-1}. HRMS *m/z* calcd for $C_{23}H_{28}N_2O_3$ 403.1998 $(M+Na)^+$, found 403.1982.

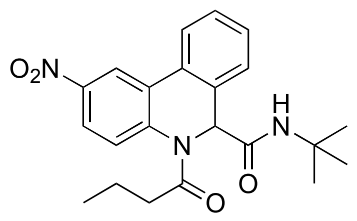

***N-tert*-butyl-5-butyryl-2-nitro-5,6-dihydrophenanthridine-6-carboxamide (1c):**

84%. Brown oil; ^{1}H NMR (300 MHz, CD_3OD, 298K) (rotamers) δ 0.74 (t, 3H, *J*= 7.0 Hz), 1.06 (s, 9H), 1.50-1.54 (m, 2H), 2.35-2.50 (m, 1H), 2.60-2.75 (m, 1H), 6.19 (s, 1H), 7.30-7.45 (m, 3H), 7.59 (d, 1H, *J*= 8.9 Hz), 7.92 (d, 1H, *J* = 7.5 Hz), 8.12 (dd, 1H, *J* = 8.9 Hz, *J*= 2.5 Hz), 8.64 (d, 1H, *J*= 2.5 Hz); ^{13}C NMR (75 MHz, CD_3OD, 298K) δ 13.8, 20.1, 28.6 (3C), 37.3, 52.5, 59.9, 120.5, 123.8, 125.1, 127.4, 128.7, 130.5 (2C), 130.6, 130.8, 135.3, 143.1, 146.8, 169.9, 175.1; IR (CH_2Cl_2) ν 3358, 3046, 2965, 1675, 1524, 1449, 1341, 1207 cm^{-1}. HRMS *m/z* calcd for $C_{22}H_{25}N_3O_4$ 418.1743 $(M+Na)^+$, found 418.1748.

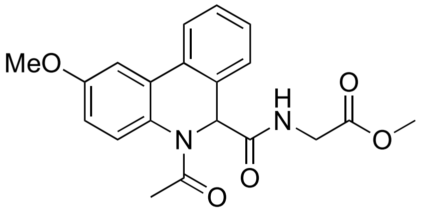

Methyl 2-(5-acetyl-5,6-dihydro-2-methoxyphenanthridine-6-carboxamido) acetate (1d):

39%. Orange oil; ^{1}H NMR (500 MHz, CD_3OD, 298K) (rotamers) δ 2.11 (s, 3H), 3.47 (s, 3H), 3.65 (d, 1H, *J*= 17.8 Hz), 3.70 (d, 1H, *J*= 17.8 Hz), 3.79 (s, 3H), 6.43 (s, 1H), 6.86 (d, 1H, *J*= 8.0 Hz), 7.30-7.40 (m, 5H), 7.7 (d, 1H, *J*= 8.0 Hz); ^{13}C NMR (125 MHz, CD_3OD, 298K) δ 22.1, 42.2, 52.5, 56.1, 58.3, 110.4, 115.0 (2C), 124.9 (2C), 128.4, 129.4, 129.9, 130.2, 132.4, 134.5, 160.0, 170.0 (2C), 172.8; IR (CH_2Cl_2) ν 3340, 2929, 1737, 1660, 1500, 1451, 1375, 1313, 1218 cm^{-1}. HRMS *m/z* calcd for $C_{20}H_{20}N_2O_5$ 391.1270 $(M+Na)^+$, found 391.1280.

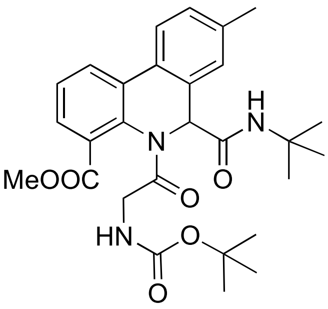

Methyl 5-(2-(*tert*-butoxycarbonylamino)acetyl)-6-(*tert*-butylcarbamoyl)-8-methyl-5,6-dihydrophenanthridine-4-carboxylate (1e):

46%. Orange oil; ^{1}H NMR (500 MHz, CD_3OD, 278K) (rotamers) δ 1.15 (s, 9H), 1.32, 1.45 (s, 9H), 2.43, 2.46 (s, 3H), 3.94 (s, 3H), 4.13 (d, 1H, *J*= 17.0 Hz), 4.25 (d, 1H, *J* = 17.0 Hz), 5.92, 5.93 (s, 1H), 7.28-7.32 (m, 1H), 7.40 (m, 1H), 7.48-7.57 (m, 1H), 7.70-7.80 (m, 1H), 8.07-8.10 (m, 1H), 8.39-8.44 (m, 1H); ^{13}C NMR (75 MHz, CD_3OD, 298K) δ 22.4, 29.8 (6C), 45.1, 54.0, 54.5, 62.4, 81.8, 125.9, 128.9, 129.0, 129.9, 130.3, 131.9, 132.5, 140.9, 170.3, 170.8, 170.9, 172.5; IR (CH_2Cl_2) ν 3321, 2963, 1708, 1672, 1521, 1453, 1392, 1367, 1272 cm^{-1}. HRMS *m/z* calcd for $C_{28}H_{35}N_3O_6$ 532.2448 $(M+Na)^+$, found 532.2408.

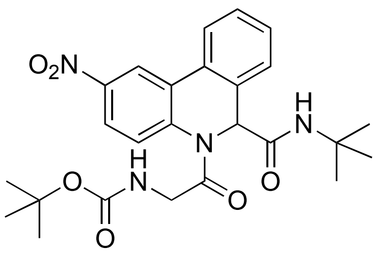

tert-butyl 2-[6-(tert-butylcarbamoyl)-2-nitrophenanthridin-5(6H)-yl]-2-oxoethylcarbamate (1f):

67%. yellow oil; ^{1}H NMR (300 MHz, CD_3OD, 298K) δ 1.07 (s, 9H), 1.29 (s, 9H), 3.84 (d, 1H, *J*= 17.0 Hz), 4.27 (d, 1H, *J*= 17.0 Hz), 6.13 (s, 1H), 6.52 (d, 1H, *J*= 9.0 Hz), 7.33-7.45 (m, 2H), 7.75 (d, 1H, *J*= 9.0 Hz), 7.86-7.93 (m, 2H), 8.13 (dd, 1H, *J*= 9.0 Hz, *J*= 2.5 Hz), 8.65 (d, 1H, *J*= 2.5 Hz); ^{13}C NMR (75 MHz, $CDCl_3$, 298K) δ 28.3 (3C), 28.4 (3C), 40.9, 52.0, 59.0, 80.5, 113.3, 119.8, 123.3, 124.3, 125.7, 128.0, 129.2, 129.7, 129.8, 132.8, 139.7, 145.7, 156.0, 166.0, 169.4; IR (CH_2Cl_2) ν 3337, 2970, 1680, 1516, 1446, 1336, 1218, 1159 cm^{-1}. HRMS *m/z* calcd for $C_{25}H_{30}N_4O_6$ 505.2063 $(M+Na)^+$, found 505.2054.

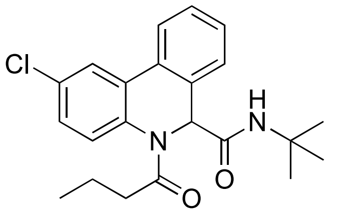

***N-tert*-butyl-5-butyryl-2-chloro-5,6-dihydrophenanthridine-6-carboxamide (1g):**

74%. brown oil; ^{1}H NMR (300 MHz, $CDCl_3$, 298K) δ 0.78 (t, 3H, *J*= 7.0 Hz), 1.07 (s, 9H), 1.45-1.70 (m, 2H), 2.34-2.38 (m, 1H), 2.52-2.56 (m, 1H), 5.64, 6.18 (2 brs, 1H), 7.08-7.24 (m, 1H), 7.22 (dd, 1H, *J*= 8.0 Hz, *J*= 2.0 Hz), 7.30-7.40 (m, 4H), 7.71 (d, 1H, *J*= 2.0 Hz); ^{13}C NMR (75 MHz, $CDCl_3$, 298K) (rotamers) δ 13.7, 19.2, 28.5 (3C), 35.9, 51.5, 123.6, 124.7 & 124.8, 126.3 & 126.3, 127.8 (2C), 128.4, 128.9 (2C), 129.0, 129.7, 132.1, 133.6, 167.3, 173.3 &

173.3; IR (CH_2Cl_2) ν 3353, 2965, 1672, 1516, 1489, 1384, 1215 cm^{-1}. HRMS *m/z* calcd for $C_{22}H_{25}ClN_2O_2$ 407.1502 $(M+Na)^+$, found 407.1491.

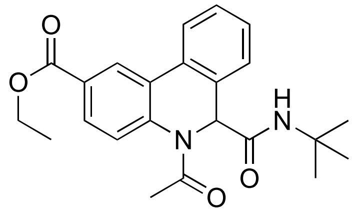

Ethyl 5-acetyl-6-(*tert*-butylcarbamoyl)-5,6-dihydrophenanthridine-2-carboxylate (1h):

93%. yellow oil; ^1H NMR (300 MHz, CD_3OD, 298K) (rotamers) δ 1.24 (s, 9H), 1.34 (t, 3H, *J*= 7.0 Hz), 2.05 (s, 3H), 4.31 & 4.46 (q, 2H, *J*= 7.0 Hz), 6.22 (bs, 1H), 7.33 (dd, 1H, *J*= 8.0 Hz, *J*= 2.1 Hz), 7.36-7.44 (m, 1H), 7.50 (d, 1H, *J*= 8.2 Hz), 7.67 (d, 1H, *J*= 8.0 Hz), 7.85 (dd, 1H, *J*= 7.4 Hz, *J*= 2.0 Hz), 7.91 (dd, 1H, *J*= 8.0 Hz, *J*= 2.0 Hz), 8.41 (d, 1H, *J*= 2.0 Hz); ^{13}C NMR (75 MHz, CD_3OD, 298K) δ 13.2, 21.3, 27.3 (3C), 52.3, 61.01, 79.2, 123.4, 125.1, 127.7, 128.4, 128.6, 128.9, 129.3, 130.2, 135.0, 136.0, 141.4, 145.9, 167.4, 169.9, 172.5; IR (CH_2Cl_2) ν 3668, 3351, 2971, 1716, 1666, 1607, 1451, 1368, 1252 cm^{-1}. HRMS *m/z* calcd for $C_{23}H_{26}N_2O_4$ 417.1790 $(M+Na)^+$, found 417.1798.

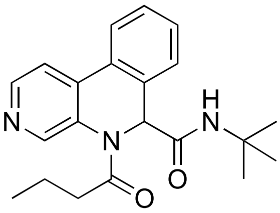

***N*-tert-butyl-5-butyryl-5,6-dihydrobenzo[c][1,7]naphthyridine-6-carboxamide (1i):**

81%. colorless oil; ^1H NMR (500 MHz, $CDCl_3$, 293K) δ 0.80-0.90 (m, 3H), 1.11 (s, 9H), 1.40-1.70 (m, 2H), 2.37-2.56 (m, 1H), 2.75-2.86 (m, 1H), 5.80 (s, 1H), 6.17 (brs, 1H), 7.37 (d, 1H, *J*= 7.3 Hz), 7.40-7.48 (m, 2H), 7.71 (d, 1H, *J*= 7.6 Hz), 7.81 (t, 1H, *J*= 7.3 Hz), 8.45 (d, 1H, *J*= 7.6 Hz), 8.56 (brs, 1H); ^{13}C NMR (75 MHz, $CDCl_3$, 293K) δ 13.6, 19.1, 28.6 (3C), 35.7, 51.7, 118.5 (2C), 124.3,

128.7 (2C), 129.3, 130.8, 134.9, 144.2, 173.1; IR (CH_2Cl_2) ν 3225, 2910, 1672, 1502, 1451, 1215 cm^{-1}. HRMS *m/z* calcd for $C_{21}H_{25}N_3O_2$ 374.1844 (M+Na)+, found 374.1821.

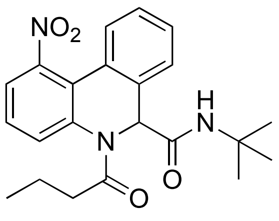

1j-a

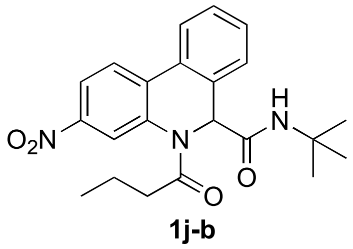

1j-b

***N*-tert-butyl-5-butyryl-1-nitro-5,6-dihydrophenanthridine-6-carboxamide (1j-a) and *N*-tert-butyl-5-butyryl-3-nitro-5,6-dihydrophenanthridine-6-carboxamide (1j-b):**

85% (**1j-a**: 45% and **1j-b**: 40 %).

1j-a: orange oil; ^{1}H NMR (300 MHz, $(CD_3)_2SO$, 298K) δ 0.70-0.75 (m, 3H), 1.05 (s, 9H), 1.34-1.58 (m, 2H), 2.30-2.50 (m, 1H), 2.60-2.74 (m, 1H), 6.22 (brs, 1H), 7.26 (d, 1H, *J*= 7.5 Hz), 7.32 (s, 1H), 7.37 (t, 1H, *J*= 7.5 Hz), 7.43 (t, 1H, *J*= 7.5 Hz), 7.58 (t, 1H, *J*= 8.1 Hz), 7.68 (d, 1H, *J*= 7.5 Hz), 7.72 (d, 1H, *J*= 8.1 Hz), 7.90 (d, 1H, *J*= 8.1 Hz); ^{13}C NMR (75 MHz, $CDCl_3$, 298K) δ 13.7, 18.7, 28.1 (3C), 35.7, 51.9, 68.5, 121.0, 121.1, 126.5, 127.5, 127.6, 128.3 (2C), 129.5, 129.6, 129.7 (2C), 129.8, 166.4, 171.9; IR (CH_2Cl_2) ν 3411, 2963, 1666, 1524, 1441, 1361, 1211 cm^{-1}. HRMS *m/z* calcd for $C_{22}H_{25}N_3O_4$ 418.1743 $(M+Na)^+$, found 418.1718.

1j-b: yellow oil; ^{1}H NMR (300 MHz, $(CD_3)_2SO$, 298K) δ 0.80-0.85 (m, 3H), 1.09 (s, 9H), 1.48-1.64 (m, 2H), 2.40-2.56 (m, 1H), 2.72-2.80 (m, 1H), 6.23 (brs, 1H), 7.48 (m, 1H), 7.49 (d, 1H, *J*= 5.5 Hz), 7.66-7.70 (m, 1H), 7.74 (s, 1H), 8.00-8.05 (m, 1H), 8.08 (dd, 1H, *J*= 8.5 Hz and *J*= 2.1 Hz), 8.17 (d, 1H, *J*= 8.5 Hz), 8.45 (bs, 1H); ^{13}C NMR (75 MHz, $CDCl_3$, 298K) δ 13.7, 19.2, 28.6 (3C), 35.8, 51.7, 58.3, 120.0, 121.1, 124.5, 125.3, 128.4, 129.2, 129.3, 130.1, 134.2, 135.8, 146.8, 150.2, 166.8, 173.2; IR (CH_2Cl_2) ν 3333, 2965, 1672, 1522, 1342,

1214 cm^{-1}. HRMS *m/z* calcd for $C_{22}H_{25}N_3O_4$ 418.1743 $(M+Na)^+$, found 418.1750.

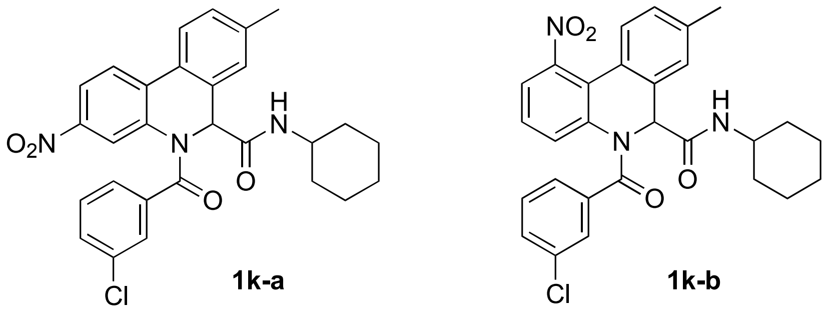

1k-a **1k-b**

5-(3-chlorobenzoyl)-*N*-cyclohexyl-8-methyl-3-nitro-5,6-dihydrophenanthridine-6-carboxamide (1k-a) and 5-(3-chlorobenzoyl)-*N*-cyclohexyl-8-methyl-1-nitro-5,6-dihydrophenanthridine-6-carboxamide (1k-b):

88% (**1k-a**: 27% and **1k-b**: 61%).

1k-a. yellow oil; ^{1}H NMR (500 MHz, $(CD_3)_2SO$, 298K) δ 1.15-1.60 (m, 10H), 2.35, 2.37 (s, 3H), 3.35-3.40 (m, 1H), 6.04 (brs, 1H), 7.07 (brs, 1H), 7.20-7.32 (m, 4H), 7.37 (t, 1H, *J*= 8.0 Hz), 7.51-7.60 (m, 4H), 7.84 (d, 1H, *J*= 8.0 Hz); ^{13}C NMR (75 MHz, $CDCl_3$, 298K) δ 21.4, 24.5, 24.6, 25.3, 32.4, 32.6, 48.7, 59.8, 121.2, 121.8, 124.2, 126.5, 127.1, 127.7, 128.1, 129.2, 129.6, 129.8, 130.5, 131.5, 133.4, 134.8, 135.4, 138.0, 140.5, 148.1, 165.9, 168.0; IR (CH_2Cl_2) ν 3341, 2924, 1662, 1525, 1464, 1364, 1323, 1282 cm^{-1}. HRMS *m/z* calcd for $C_{28}H_{26}ClN_3O_4$ 526.1510 $(M+Na)^+$, found 526.1480.

1k-b: brown oil; ^{1}H NMR (500 MHz, $(CD_3)_2SO$, 298K) δ 1.05-1.20 and 1.45-1.65 (m, 10H), 2.37 (s, 3H), 3.25-3.35 (m, 1H), 5.97 (brs, 1H), 7.22-7.24 (m, 3H), 7.34 (d, 1H, *J*= 8.0 Hz), 7.41 (t, 1H, *J*= 7.5 Hz), 7.54 (s, 1H), 7.58 (d, 1H, *J*= 8.0 Hz), 7.98 (d, 1H, *J*= 8.0 Hz), 8.01 (dd, 1H, *J*= 9.0 Hz, *J*= 2.1 Hz), 8.04 (d, 1H, *J*= 9.0 Hz), 8.15 (d, 1H, *J*= 9.0 Hz); ^{13}C NMR (125 MHz, $CDCl_3$, 298K) δ 21.5, 24.5 (2C), 25.4, 32.6, 32.8, 48.6, 59.7, 120.7, 120.8, 124.6, 124.7, 126.6, 127.3, 128.6, 129.4, 129.5, 130.5, 132.0, 133.3, 133.7, 134.8, 134.9, 136.0,

141.0, 146.2, 166.5, 168.8; IR (CH_2Cl_2) ν 3379, 2926, 1658, 1648, 1523, 1342 cm^{-1}. HRMS *m/z* calcd for $C_{28}H_{26}ClN_3O_4$ 526.1510 $(M+Na)^+$, found 526.1503.

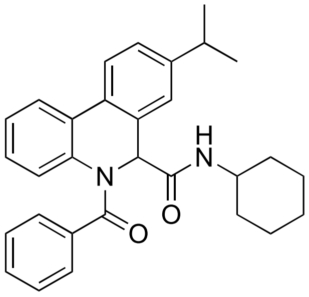

5-benzoyl-*N*-cyclohexyl-8-*iso*-propyl-5,6-dihydrophenanthridine-6-carboxamide (1l):

80%. orange oil; ^1H NMR (300 MHz, CD_3OD, 298K) δ 1.10-1.30 and 1.55-1.70 (m, 10H), 1.37 (d, 6H, *J*= 7.0 Hz), 3.06 (m, 1H), 3.50 (m, 1H), 6.24 (s, 1H), 6.78 (d, 1H, *J*= 8.1 Hz), 6.97 (td, 1H, *J*= 8.1 Hz, *J*= 1.2 Hz), 7.21 (td, 1H, *J*= 8.1 Hz, *J*= 1.1 Hz), 7.26-7.38 (m, 6H), 7.40-7.45 (m, 2H), 7.84 (d, 1H, *J*= 1.5 Hz), 7.90 (dd, 1H, *J*= 8.0 Hz, *J* = 1.5 Hz); ^{13}C NMR (75 MHz, $CDCl_3$, 298K) δ 24.5, 24.6, 25.9 (2C), 26.5, 33.4, 35. 6 (2C), 56.0, 60.4, 127.7, 125.3, 127.0, 127.5, 127.6, 128.5, 129.3, 129.3 (3C), 130.3 (2C), 131.9, 132.0, 132.1, 136.0, 137.8, 151.3, 170.2, 171.9; IR (CH_2Cl_2) ν 3343, 2925, 1677, 1649, 1487, 1444, 1369, 1328, 1274 cm^{-1}. HRMS *m/z* calcd for $C_{30}H_{32}N_2O_2$ 475.2361 $(M+Na)^+$, found 475.2349.
